# Editorial: Metalloenzymes: Potential Drug Targets

**DOI:** 10.3389/fphar.2021.746925

**Published:** 2021-09-24

**Authors:** Jamshed Iqbal, Claus Jacob, Jean Sévigny

**Affiliations:** ^1^ Centre for Advanced Drug Research, COMSATS University Islamabad, Abbottabad, Pakistan; ^2^ Division of Bioorganic Chemistry, School of Pharmacy, University of Saarland, Saarbruecken, Germany; ^3^ Centre de Recherche du CHU de Québec - Université Laval, Québec City, QC, Canada; ^4^ Département de Microbiologie-Infectiologie et d’Immunologie, Faculté de Médecine, Université Laval, Québec City, QC, Canada

**Keywords:** metalloenzymes, ecto-5′-nucleotidase, nucleotide pyrophosphatase/phosphodiesterases, nucleoside triphosphate diphosphohydrolases, inhibitors

Metalloenzymes have an important role in the regulation of many biological functions. Overexpressed and/or reduced secretion of such enzymes lead to different complications of clinical interest. The metal ions present in enzymes control the structure, folding, and functions of such proteins. The protein data bank (PDB) revealed that over 50% of proteins contain metal ions (Solomon et al., 1996). The development of metalloenzyme inhibitors are of interest in the treatment of various diseases. The interaction of ligands, i.e., compounds as inhibitors with target proteins via active sites provide a means of curing diseases. Most aptly, the inhibitors reported by academic or pharmaceutical usage of small molecules as inhibitors provide a rapid and viable way to treat diseases. Urease is a ubiquitous metalloenzyme, produced by various cell types from plants, fungi, and bacteria, etc., that bears a nickel atom in its active pocket. It hydrolyzes the urea into ammonia and carbamate which further decompose to ammonia and CO_2_. The overexpression of urease was known to be linked with ulcers, hepatic coma, and formation of urinary stones (Upadhyay, 2012; Kappaun et al., 2018).

Carbonic anhydrase with zinc metal ion catalyze the hydration of CO_2_ with water to produce hydrogen carbonate and H^+^ ions (Alvarez-Leefmans and Delpire, 2009). The hydration reaction involves the nucleophilic attack of the metal-bounded hydroxy (OH) group with the carbon (C) atom of carbon dioxide species (Silverman and Lindskog, 1988). The coordination of carbonic anhydrases (CAs) with metal ion occurs at active sites via binding with histidine, cysteine, and/or glutamine residues to form a tetrahedral shape (Steiner et al., 1975). The inhibitors of CAs have been employed as diuretic and antiglaucoma agents as well as anti-obesity and anticancer agents (Supuran, 2008).

Furthermore, ubiquitous ecto-nucleotidases such as 1) nucleoside triphosphate diphosphohydrolases (NTPDases), 2) nucleotide pyrophosphatase/phosphodiesterases (NPPs), 3) alkaline phosphatases (APs or ALPs), and 4) ecto-5′-nucleotidase (e5′NT) are all responsible for the integrity of proper cell functioning (Supuran, 2008). The NPPs possess zinc (Zn^2+^) metal ion at active sites while the e5′NT has additionally magnesium ion (Mg^2+^) at the active site. The overexpression of surface-located ecto-enzymes hydrolyzing nucleotides causes various complications which affect different functions such as cell proliferation, apoptosis as well as degenerative, neurological, and immunological responses. In the current issue, Baqi et al. reported the use of anthraquinone derivatives as NTPDase inhibitors which showed selectivity towards NTPDase2 and -3. The compound, 1-amino-4-(9-phenanthrylamino)-9,10-dioxo-9,10-dihydroanthracene-2-sulfonate, with an IC_50_ value of 539 nM was found to be a potent inhibitor of NTPDase2, while the anthraquinone, 1-amino-4-[3-(4,6-dichlorotriazin-2-ylamino)-4-sulfophenylamino]-9,10-dioxo-9,10-dihydroanthracene-2-sulfonate, showed potency and selectivity towards NTPDase3 with an IC_50_ of 390 nM. The most potent compounds could serve as front-runners in the goal of treating different pathological complications (Baqi et al.). The inhibitors of NTPDase1 may serve as potential leads in cancer therapy. Adenosine derivatives were screened for CD39 and CD73. The biological assay results showed selective CD39 and dual CD39/CD73 inhibitors (Schäkel et al.). A docking study further showed putative binding of nucleotide analogs with target enzymes. Thus, the inhibition of metalloenzymes might be useful to cure different clinically important complications.

Prof. Iqbal’s research group have made significant progress in the development and identification of different compounds as inhibitors of metalloenzymes. They have reported several inhibitors of carbonic anhydrase activity such as sulfonamides, sulfonates, and sulfamate derivatives (Zaraei et al., 2019; Saeed et al., 2021). Furthermore, many other types of molecules have also been reported as carbonic anhydrase inhibitors (Al-Rashida et al., 2014; Saeed et al., 2014; Zaib et al., 2014; Al-Rashida et al., 2015; Saeed et al., 2017; Abbas et al., 2018; Abbas A. et al., 2019). Supuran (2008) published an excellent review in *Nature Review Drug Discovery* to present carbonic anhydrase and its inhibitors. The presence of zinc metal provides a means of binding enzymes with several classes of compounds including sulphonamides, sulphamates, and sulphamides.

Urease is also a common target for treating ulcers which possesses two nickel atoms in its core structure. The inhibition of urease has been reported by using different heterocycles. In their studies, Iqbal et al. have reported 1,3-thiazoles (Channar et al., 2021), benzohydrazide derivatives (Abbas S. et al., 2019), acridine-based (thio)semicarbazones, hydrazones (Isaac et al., 2019), and semicarbazones derivatives (Qazi et al., 2018) as urease inhibitors. The copper metal ion containing tyrosinase has been known to be involved in melanin biosynthesis. Lavinda et al. developed a 3D model of the structure of human tyrosinase and TYRP2 on the basis of their crystallographic structure. The mechanism of mercury chloride (HgCl_2_)-induced tyrosinase inactivation was investigated in this study (Chen et al.).

Iqbal et al. have also made progress on the inhibition of other biological targets which include other ecto-nucleotidases. Various types of heterocyclic scaffolds such as the inhibitors of nucleotide pyrophosphatase/phosphodiesterases (NPPs) include substituted trifluoromethyl quinoline (Semreen et al., 2019), arylated thiadiazolopyrimidones (Jafari et al., 2016), and *p*-nitrophenyl thymidine 5- monophosphate (Raza et al., 2011). Moreover, the development of ecto-5′-nucleotidase inhibitors may also serve to treat cancer (Iqbal et al., 2013). The heterocyclic compounds which have been synthesized and screened as NTPDases include oxoindolin phenylhydrazine carboxamides (Afzal et al., 2021), pyrrolo[2,3-b]pyridine derivatives (Ullah et al., 2021), spirooxindole derivatives (Ashraf et al., 2020), sulfonylhydrazones (Younus et al., 2020), 2-substituted-7-trifluoromethyl-thiadiazolopyrimidones (Afzal et al., 2020), pyrazolyl pyrimidinetrione, and thioxopyrimidinedione conjugates as selective inhibitors of human ectonucleotidase (Andleeb et al., 2019), etc. Furthermore, the review articles of Iqbal (Iqbal, 2019) have also reported the importance of ectonucleotidase inhibitors in the treatment of various diseases. Though there are many other metal ions-containing proteins which have been targeted to treat clinically relevant conditions, Iqbal et al. have made a significant scientific contribution in finding good choices in the inhibition of urease, carbonic anhydrase, and ectonucleotidases.

In summary, metalloproteins present relevant targets for the development of modulators in order to treat various types of diseases. In this view, development of metalloprotein inhibitors with different structures, such as heterocyclic compounds, provide an excellent opportunity to treat such diseases. The presence of metal ions in these proteins help them bind with the inhibitor and play an important role in the inhibition process. Libraries of small heterocyclic molecules can be readily prepared to screen them against various target proteins to cure corresponding diseases.
